# A murine model demonstrating reversal of structural and functional correlates of cirrhosis with progenitor cell transplantation

**DOI:** 10.1038/s41598-019-51189-7

**Published:** 2019-10-28

**Authors:** Mark D. Muthiah, Daniel Q. Huang, Lei Zhou, Nur Halisah Jumat, Mahesh Choolani, Jerry Kok Yen Chan, Aileen Wee, Seng Gee Lim, Yock-Young Dan

**Affiliations:** 1 Department of Gastroenterology and Hepatology, National University Health System, Singapore, Singapore; 2 Department of Medicine, Yong Loo Lin School of Medicine, National University of Singapore, Singapore, Singapore; 3 Department of Obstetrics and Gynaecology, National University Health System, Singapore, Singapore; 4 Department of Reproductive Medicine, KK Women’s and Children’s Hospital, Singapore, Singapore; 5 Experimental Fetal Medicine Group, Yong Loo Lin School of Medicine, National University of Singapore, Singapore, Singapore; 6 Department of Pathology, National University Health System, Singapore, Singapore

**Keywords:** Liver cirrhosis, Liver fibrosis

## Abstract

Development of cell transplantation for treating liver cirrhosis hinges critically on the availability of animal models for studying human stem cell transplantation. We report an immune-permissive murine model of liver cirrhosis with full clinical correlates of decompensated liver disease, and allows testing efficacy of stem cell transplantation. Liver cirrhosis was induced in Nod-scid gamma(NSG) mice with oral thioacetamide(TA) and compared to controls over 12 months. 4 month TA treated cirrhotic mice were then transplanted intrasplenically with 2million human fetal liver progenitor cells(HFH) and compared with cirrhotic controls 2 months after transplantation. NSG-TA mice developed shrunken and nodular livers with histological evidence of fibrosis as compared to controls. This was associated with evidence of worsening decompensated liver disease, with jaundice, hypoalbuminemia, coagulopathy, and encephalopathy in NSG-TA mice. Transplantation of HFH resulted in improvement in both fibrosis and markers of decompensated liver disease. We have demonstrated that NSG-TA mice can recapitulate the full clinical picture of structural and functional cirrhosis, both of which can be improved by transplantation of human fetal liver cells. This model serves as a valuable tool for validation of *in vivo* liver stem cell transplantation and opens up opportunities for studying the mechanism how stem cells reverse fibrosis.

## Introduction

Liver cirrhosis is a major cause of mortality and morbidity in the world today. As the final common pathway in almost all chronic liver diseases^1,2^, its prevalence is estimated at about 1% worldwide^3^ and mortality due to liver cirrhosis is rapidly rising in developed countries^4^. Although liver transplantation is a curative treatment for liver cirrhosis^3^, the growing number of potential transplant recipients on the waiting lists has outstripped the supply of donor organs^5^, with many patients dying while on the waiting list.

Cirrhosis is the final common pathway for many chronic liver diseases including viral, metabolic and toxic causes. The persistent injury to the liver results in continual cell death and triggering of chronic inflammatory mediators. These activate hepatic stellate cells to become myofibroblasts which secrete extracellular matrices and lay down collagen scars within the parenchyma^6^. This leads to further loss of hepatocytes, nodular scar tissue formation and increase in sinusoidal resistance. Three broad sequelae ensue: (i) deficiency in hepatocytic function resulting in hypoalbuminemia, hyperbilirubinemia and coagulopathy; (ii) portal hypertension leading to bleeding varices, ascites formation and encephalopathy; and (iii) increased risk of development of hepatocellular carcinoma^3^.

Much effort has gone into attempting to understand the pathogenesis of liver cirrhosis in the hope of identifying novel targets or applications for therapies. Advances in anti-fibrotic therapies have seen scores of potential agents being tested in different phases of clinical trials^7^. An alternative approach seeks to harvest and transplant liver stem cells into the cirrhotic livers in an attempt to repair the liver and augment liver insufficiency^8,9^. Progress along both prongs of approach however, has been slow. This is in part due to the lack of a good small animal model that replicates the full pathophysiology and clinical correlates of decompensated cirrhosis for testing efficacy and safety^10^.

Current murine models of cirrhosis include common bile duct ligation, or hepatotoxin administration - alpha-naphthyl-isothiocyanate (ANIT), allyl alcohol (AA), carbon tetrachloride (CCl4), 3,5-diethoxycarbonyl-1,4-dihydrocollidine (DDC) and silica^11^. These models of chronic liver disease do develop fibrosis and nodularity of the liver but few models develop full blown cirrhosis or clinical complications that recapitulate the full clinical spectrum of decompensated liver disease in human patients. While in principle, efficacy for progenitor cell transplantation has been shown in *in vivo* models such as fumarylacetoacetate hydrolase knockout (FAH), urokinase-type plasminogen activator overexpression (uPA) and *AhCre*, these models use genetic manipulation to exert extreme levels of survival selection pressures to achieve high degrees of stem cell or progenitor cell repopulation^12–14^. They also do not simulate the clinical complications of a decompensated cirrhotic patient. The relevance of these models to clinical cirrhosis and the real life efficacy of new potential therapies in reversing the complications of cirrhosis are not known.

We report an immune-permissive murine model of liver fibrosis using thioacetamide (TA) in NOD Scid Gamma (NSG) mice that demonstrates the full structural and clinical correlates of decompensated liver disease and allows testing of efficacy of liver stem cell transplantation. TA has been shown to produce stable liver fibrosis, which is similar in clinical progression to fibrosis in humans^15^. To demonstrate principle of proof of efficacy of stem cell therapy in reversing cirrhosis, we transplanted human fetal hepatocytes (HFH), to demonstrate reversal of structural, functional and clinical correlates of liver cirrhosis.

## Materials and Methods

### Animals

All animals were housed in the National University of Singapore (NUS) animal holding unit and procedures obtained Institutional Animal Care and Use Committee (IACUC) approval. All experimental procedures were performed in accordance with guidelines and regulations from the IACUC. 3-month-old female NSG mice - (NOD.Cg-*Prkdc*^*scid*^ *Il2rg*^*tm1Wjl*^/SzJ) - were used as an immune-permissive mouse strain. C57BL/6 mice were used to validate the model of liver cirrhosis in immuno-competent mice.

### Induction of cirrhosis

**Figure 1:**
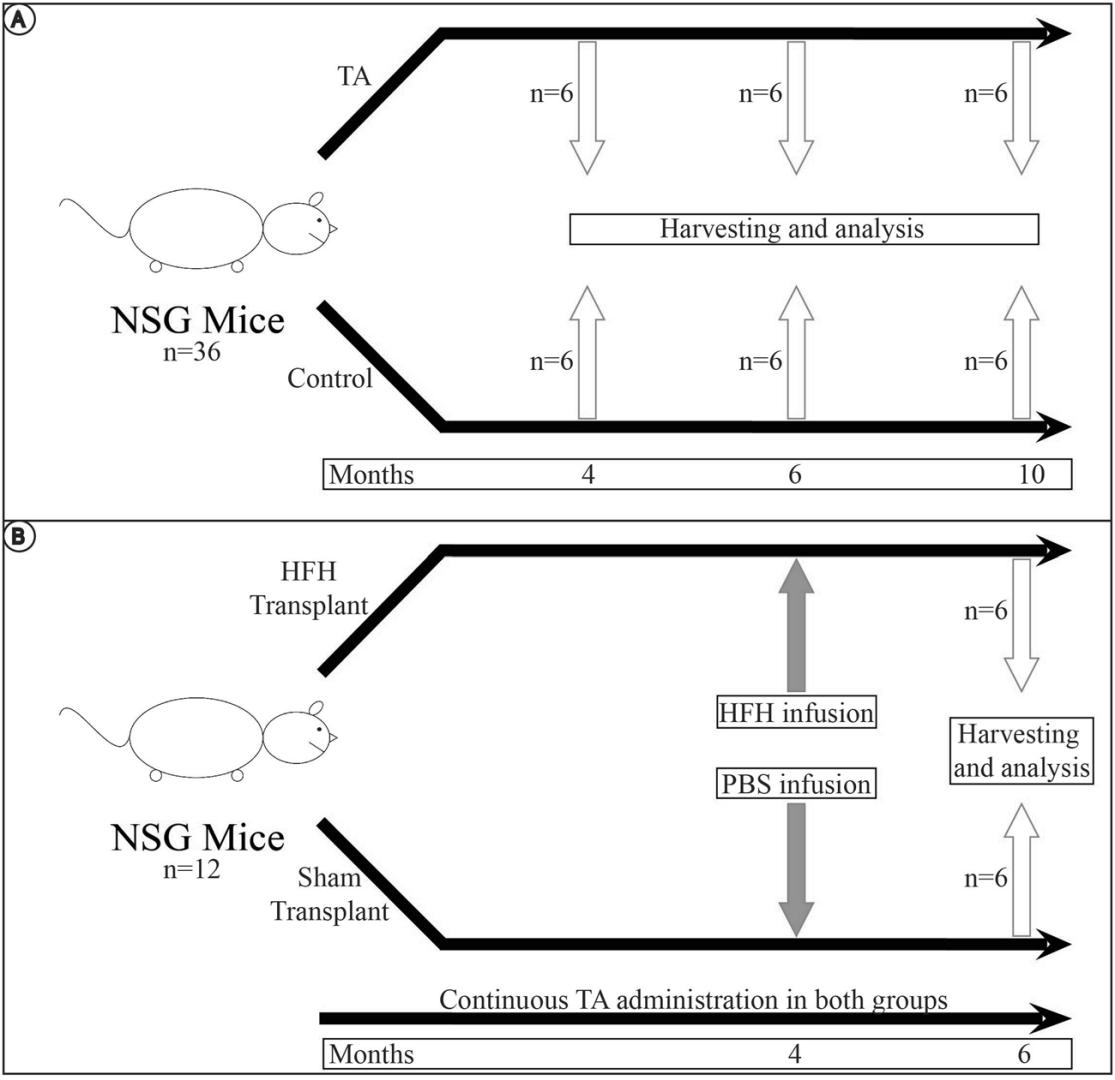
Overall conduct of experiment. (**A**) For the first part of the experiment, 18 NSG mice were fed oral TA to induce liver cirrhosis. TA was administered, and 6 mice were sacrificed at 4 months, 6 months and 10 months respectively. They were compared to 18 control NSG mice, each given normal drinking water for the same duration. (**B**) For the second part of the experiment, NSG mice were given 4 months of oral TA. They were then subsequently transplanted with intrasplenic HFH, and given a further 2 months of TA, for a total duration of 6 months of TA. These mice were sacrificed at 6 months for analysis. They were compared to sham transplant mice given oral TA for 4 months, intrasplenic phosphate buffered saline (PBS), and a further 2 months of oral TA.

Thioacetamide (TA; 200 μg/L; Sigma-aldrich) was dissolved in drinking water for administration to NSG mice. Control mice were fed with plain drinking water. Mice were sacrificed at 4 months, 6 months, and 10 months of administration of TA and compared to controls. 6 mice were used per time point in each experimental arm (Fig. 1).

### Fibrosis

Formalin-fixed, paraffin embedded histological sections of livers were cut at 4 microns thickness and stained with Sirius Red (SR), Masson Tricrhome (MT), and Hematoxylin and Eosin (H&E). Fibroblast activating protein (FAP) and anti-smooth muscle actin (aSMA) immunohistochemistry (IHC) was performed. Fibrosis was quantified using ImageJ software on 6 random areas at 10x magnification. RNA expression for Col1α1, TIMP-1 and α-SMA by real time polymerase chain reaction (RT-qPCR) was done to evaluate presence of fibrosis. Enzyme linked immunosorbent assay (ELISA) for serum liver pro-collagen was also measured.

### Clinical correlates of cirrhosis

Serum albumin, bilirubin, and prothrombin time (PT) were measured from the mice upon sacrifice, as a service at the Veterinary Diagnostic Laboratory of the National University of Singapore (NUS).

The presence of ascites was also noted.

Encephalopathy in the mice was measured using reported and validated tests including novel object recognition employing hole board testing^16^, and object and spatial recognition tests^17^.

### Human fetal liver progenitor cell isolation

The experimental protocols were approved by Institutional Review Boards from both the National University Hospital (NUH) and Kandang Kerbau Women’s and Children’s Hospital (KKWCH) – The National Healthcare Group Domain Specific Review Board (NHG DSRB), and the SingHealth Centralised Institutional Review Board (CIRB). Human fetal livers between 12 and 18 weeks gestation were obtained from NUH and KKWCH in Singapore in accordance with protocols approved by their respective Institutional Review Boards. Informed consent was obtained from the mother’s prior to using the cells from the human fetal livers. Primary fetal hepatocyte cultures were isolated and frozen as described^18^. HFH used were a heterogenous population with 80% hepatoblasts, and 20% mesenchymal cells^19^.

### Transplantation of HFH

Prior to transplantation, mice were treated for 4 months with oral TA. After cirrhosis was induced, two million HFH cells suspended in 200 μl phosphate buffered saline (PBS) were transplanted into mice intra-splenically and oral TA was continued. Negative control animals (sham transplant) were transplanted with 200 μl PBS only and oral TA was continued. Six animals were used in each experimental arm, and animals were sacrificed two months after transplantation, after being fed a total of 6 months of oral TA.

### Cell labelling and analysis

To trace the fate of the HFHs, engrafted cells were labelled with dual immunofluorescence (IF) using human specific antibodies against albumin (anti-Hu Albumin), alpha 1 antitrypsin (anti Hu A1AT), CYP2A6, and CYP3A4. The percentage of human cell engraftment was calculated based on the number of labelled human cells in 6 random portal tract areas across 20 continuous sections (50 microns apart) relative to the number of parenchymal cells. To demonstrate that the cells were mature, dual IF with human specific antibodies against albumin and AFP (anti Hu AFP) was also performed as a significant negative.

### Statistical calculations

All statistical analysis between groups were carried out using the independent samples t-test, and error bars represent standard error of n = 6 biological samples. Results were considered statistically significant when p ≤ 0.05 and analysis was done using SPSS version 20.0.

## Results

### Administration with oral TA leads to development of fibrosis

**Figure 2:**
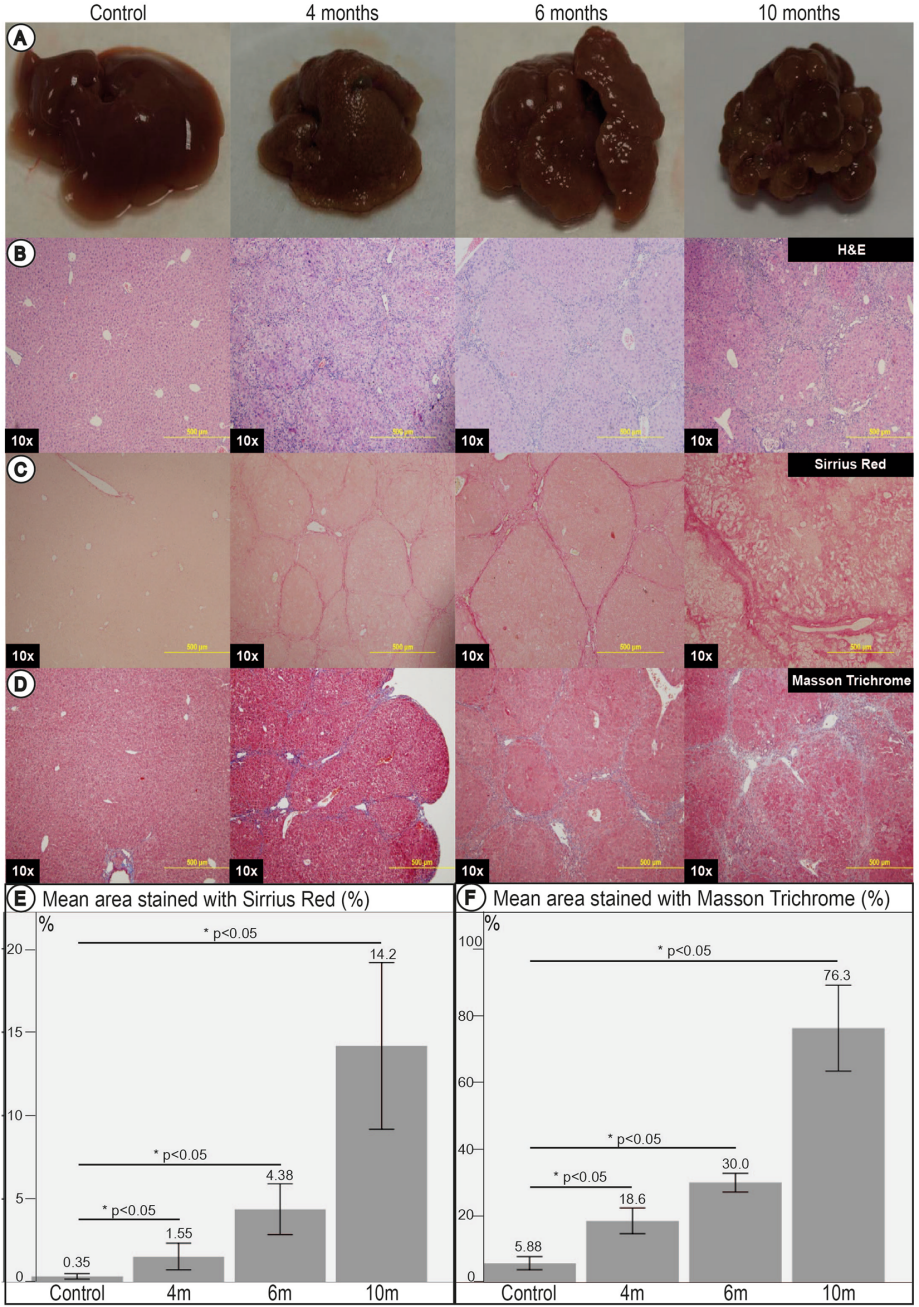
Livers and liver histology of control mice and at 4, 6 and 10 months of TA administration. (**A**) Gross livers surface of NSG mice fed with TA. Shrinkage and progressive nodularity of livers with increasing duration of TA administered was noted. By 10 months of TA administration, livers showed classical features of advanced cirrhosis. (**B**) Histological examination with H&E. (**C**) Histological examination with Sirrius Red. (**D**) Histological examination with Masson Trichrome. Stains revealed bridging fibrosis with thin septa at 4 months, consistent with mild cirrhosis. Nodule formation and broad septa were seen by 6 months, consistent with moderate cirrhosis. Architectural collapse with thick broad septa were seen at 10 months, consistent with severe cirrhosis. (**E**) Image morphometry of Sirrius Red staining. (**F**) Image morphometry of Masson Trichrome stain. These quantitatively demonstrated a significant increase in area stained with increasing duration of TA administered, further corroborating the findings on histology.

All NSG mice fed continuously with TA demonstrated gross morphology of increased surface nodularity at 4 months compared to controls. This worsened progressively with shrinkage of the liver mass at 6 months (Fig. 2A). By 10 months, the liver was significantly shrunken and showed extensive nodule formation. Control mice did not demonstrate any nodularity, and had regular sized livers across all time points (Fig. 2A).

Using Sirius Red and Masson Trichrome staining, bridging fibrosis in the liver was demonstrable by 4 months of TA treatment. Thin septa, with rounded contours and visible nodules were seen, corresponding to Laennec 4a (cirrhosis, mild, definite, or probable). Nodular fibrosis was demonstrable by 6 months, with broad septa seen, corresponding to Laennec 4b (moderate cirrhosis). By 10 months, architectural collapse with macronodule and micronodule formation was seen in keeping with advanced liver cirrhosis. There were thick broad septae seen, corresponding to Laennec 4c (severe cirrhosis) (Fig. 2B–D).

Quantitative assessment by image morphometry of collagen scars using Sirius Red and Masson Trichrome demonstrated a progressive increase in area stained with Sirius Red and Masson Trichrome (Fig. 2E,F) with prolonged administration of TA as well as compared to controls across all time points.

**Figure 3:**
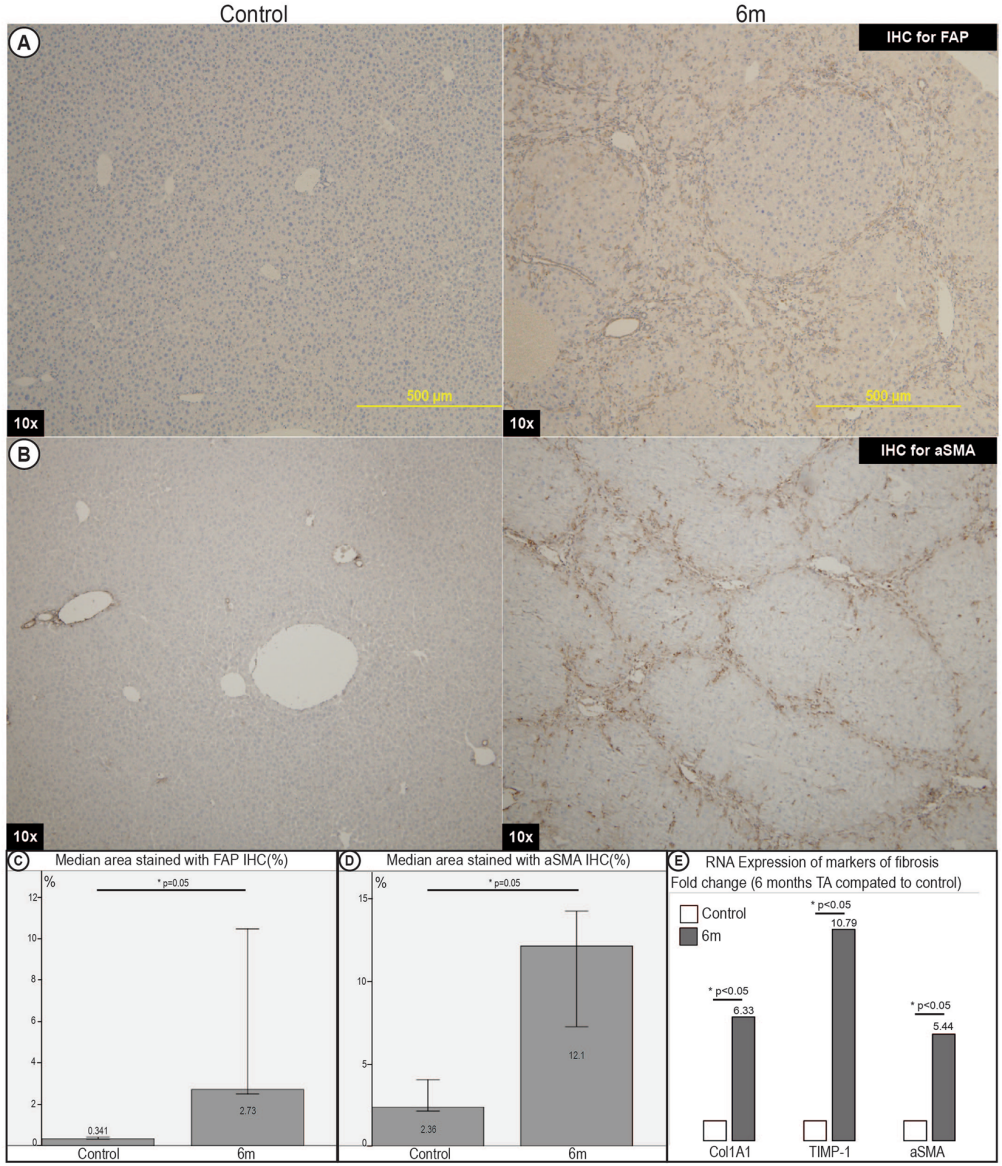
Immunohistochemistry and RT-qPCR for markers of fibrosis of livers of control mice and mice fed TA for 6 months. (**A**) Immunohistochemistry (IHC) for fibroblast activating protein (FAP). (**B**) IHC for alpha smooth muscle actin (aSMA). (**C**) Image morphometry of FAP. (**D**) Image morphometry of aSMA. Quantitative analysis showed a significant increase in the area stained positive for FAP and aSMA. This demonstrated a significant increase in markers consistent with fibroblast activation and myofibroblast activation respectively. (**E**) RT-qPCR expression compared between TA mice at 6 months and control mice for markers of fibrosis (Col1A1, TIMP-1, aSMA) demonstrated significant increase in RNA expression of markers of fibrosis.

RNA expression by RT-qPCR demonstrated increased expression of fibrosis markers of Col1α1, TIMP-1 and α-SMA in mice fed TA over 6 months as compared to control mice (Fig. 3E).

To further confirm that the scarring was consistent with activation of pathogenesis of cirrhosis, we performed immunohistochmical staining for fibroblast activating protein (FAP) as well as anti-smooth muscle actin (aSMA) to assess fibroblasts^20^ and myofibroblasts^11^ respectively. Both FAP and aSMA progressively increased in frequency in the collagen scars in line with progressive fibrosis (Fig. 3A–D).

### Mice develop functional liver insufficiency with thioacetamide

**Figure 4:**
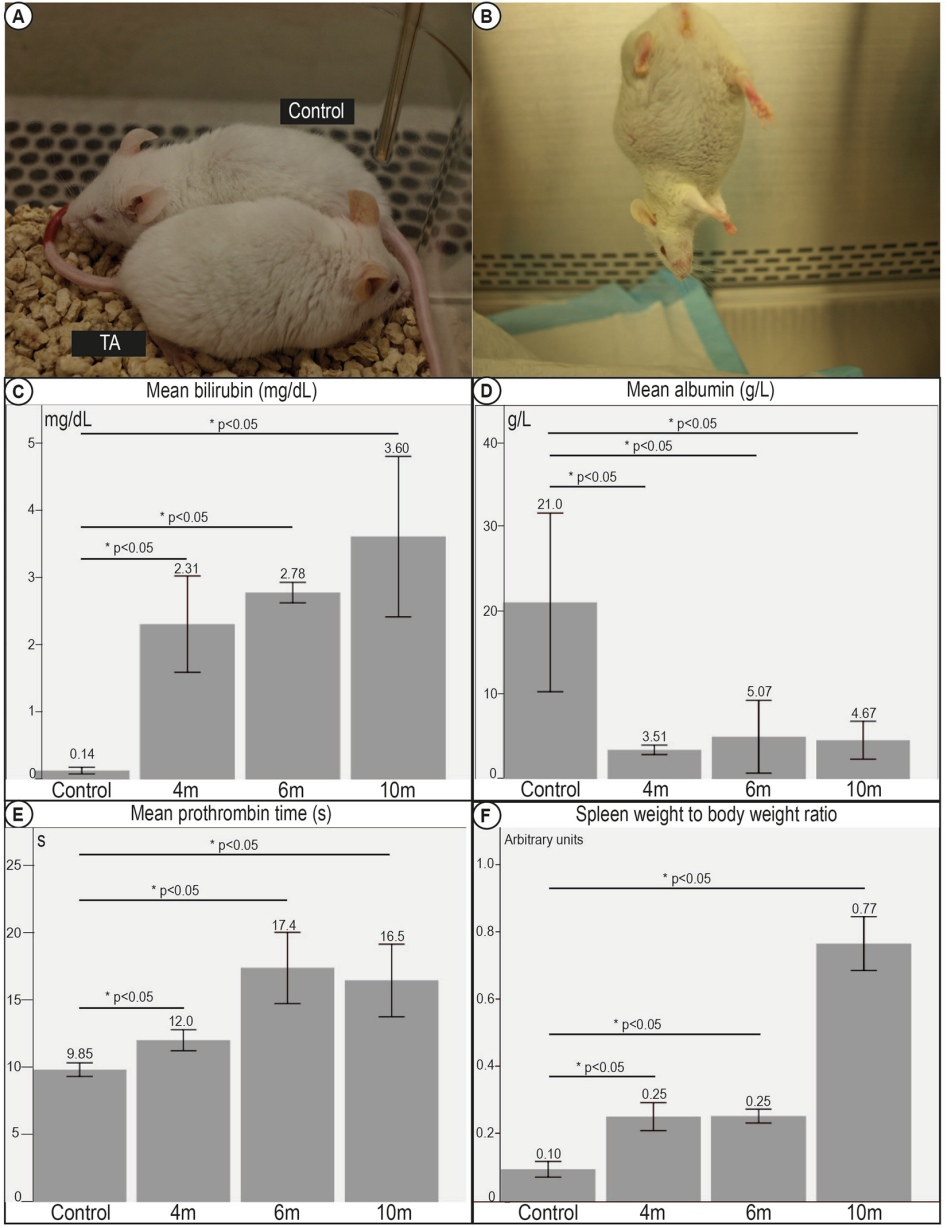
Decompensation of clinical correlates of liver cirrhosis in mice fed TA. (**A**) Mice fed TA demonstrated observable jaundice. TA fed mice had Yellowing of the ears, and appeared lethargic as compared to control mice. (**B**) All TA fed mice developed ascites by 6 months of exposure. (**C**) Serum tests revealed significant hyperbilirubinemia, (**D**) hypoalbuminemia, (**E**) coagulopathy progressing with increasing duration of TA administered, and suggesting evidence of synthetic dysfunction typical of decompensated liver disease. (**F**) Spleen weight to body weight ratios. Increase in spleen weight to body weight ratios suggesting worsening portal hypertension was seen with increasing duration of TA administered.

Mice treated with TA developed observable jaundice in the ears after being administered with TA for more than 6 months (Fig. 4A). This was consistent with a significant progressive increase in serum bilirubin across all time points as compared to control mice. The mean serum bilirubin was 0.14 (0.09–0.22) mg/dL in control mice. The bilirubin became elevated at 4 months and progressively worsened to a peak of 3.60 (1.36–4.50) mg/dL at 10 months (Fig. 4C).

This concurred with a corresponding decrease in serum albumin levels across all time points as compared to control mice with a mean nadir of 4.67 g/L at 10 months, as compared to 21.0 g/L for control mice (Fig. 4D).

To directly probe the synthetic function of the livers, we tested the prothrombin times (PT) in mouse serum as a direct measure of the clotting factors synthesised in the livers. From a baseline of 9.85 seconds in controls, coagulopathy ensued with administration of TA with the mean PT reaching a peak of 17.4 seconds at 6 months (Fig. 4E).

### Mice develop clinical decompensation

As a surrogate marker of portal hypertension, we measured the spleen size and noted significant differences in spleen weight of mice fed TA compared to controls. The spleen weights and the spleen to body weight ratios continued to increase progressively with prolonged TA and progressive fibrosis, consistent with increasing portal pressures (Fig. 4F).

On sacrificing the mice, TA fed mice after 6 months demonstrated visible ascites, while none of the control mice had any detectable ascites (Fig. 4B).

To assess if cirrhotic mice developed encephalopathy, we tested mice for attention to novel objects using validated psychometric testing. Mice fed with TA for more than 10 months paid no attention or showed no interest to a novel object placed in the enclosure, in contrast to control mice. In object and spatial recognitions tasks, cirrhotic mice with more than 10 months TA treatment took a much longer time to discover both novel and familiar objects as compared with control mice (Tables 1 and 2). No significant differences were detected between animals that received less than 8 months of TA treatment and their controls supporting the observation that encephalopathy is detectable only in late stage cirrhosis.

**Table 1:** Hole board testing.

Encephalopathy: Hole-board Testing			
Object	Time to discover (s)	Median times visited	Median time spent in each visit (s)
1-year TA	NA*	0	0
Control	444	2	2

*None of the 1-year TA mice managed to discover the object baited in the hole-board setup.

**Table 2:** Object and spatial recognition tasks.

Encephalopathy: Object and Spatial Recognition Tasks			
Object	Median time to discover novel object (s)	Median time to discover familiar object (s)	p value
1-year TA	124	111	0.05
Control	27	30	0.05

Mice fed TA for a year demonstrated poor performance in neuropsychiatric testing, consistent with encephalopathy.

5% of the mice given TA for 4 months died, while 10% of the mice given TA for 6 months died. After a year of TA treatment, 33% of mice died from end-stage liver disease.

### Administration with oral TA leads to development of hepatocellular carcinoma

**Figure 5:**
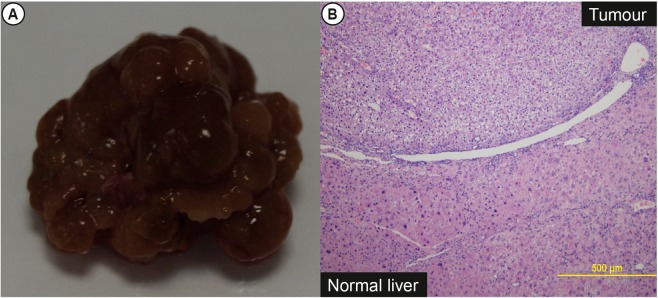
Gross images and histology of nodules in TA fed mice. (**A**) All mice fed with TA demonstrated significant surface nodularity. 2/12 of the mice fed at least 6 months TA demonstrated dominant nodules. (**B**) Histology of some of these nodules revealed trabecular disarray and nuclear atypia characteristic of hepatocellular carcinoma.

Two mice (17%) treated with TA for at least 6 months developed a large discrete tumour on the liver surface (Fig. 5A). Histological analysis of the nodules by 2 blinded liver pathologists concurred with the diagnosis of hepatocellular carcinoma based on the loss of lobular hepatic cord architecture (Fig. 5B). None of the control mice developed hepatocellular carcinoma.

### HFH transplantation demonstrated engraftment into the cirrhotic mice

**Figure 6:**
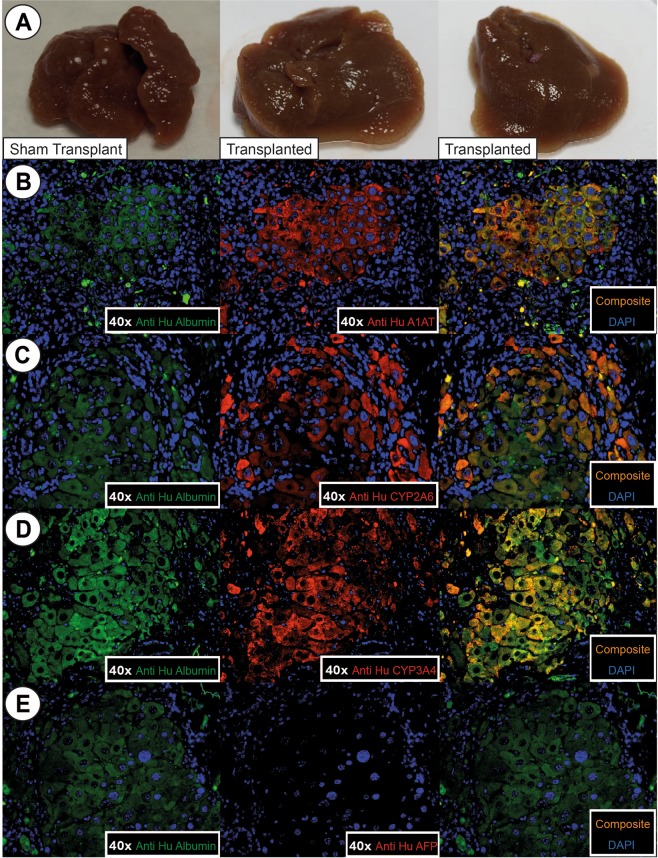
Gross liver pictures and IF of transplanted mice 2 months post-transplant. (**A**) Gross morphology of sham transplant mice livers and HFH transplanted mice livers. Transplanted mice livers were softer in texture and less nodular than livers of sham transplant mice. (**B**) Co-labelling between IF of anti human albumin and anti human alpha1-antitrypsin (A1AT), (**C**) CYYP2A6, (**D**) CYP3A4, and (**E**) absence of co-localization between anti human albumin and AFP. This demonstrated that the engrafted hepatocytes matured, and demonstrated structural and functional viability up to 2 months post-transplant. No immature hepatocytes were seen.

To demonstrate proof of principle of efficacy in stem cell transplantation, we transplanted 2 million HFH intra-splenically into cirrhotic animals treated with 6 months of TA and followed them up for 2 months. To look for engraftment, mice livers were stained for human specific albumin antibody (anti-Hu albumin), human specific alpha 1 antitrypsin (anti Hu A1AT), CYP2A6, and CYP3A4. Up to 2% of hepatocytes expressed these stains, indicating successful engraftment of functional human cells in cirrhotic mouse livers (Fig. 6B–D).

Staining with human alphafetoprotein (AFP) was negative, demonstrating that these engrafted cells were mature.

### HFH transplantation demonstrated improvement of liver fibrosis

Grossly, livers of animal transplanted with HFH were softer in texture and significantly less nodular than sham transplant mice (Fig. 6A).

**Figure 7:**
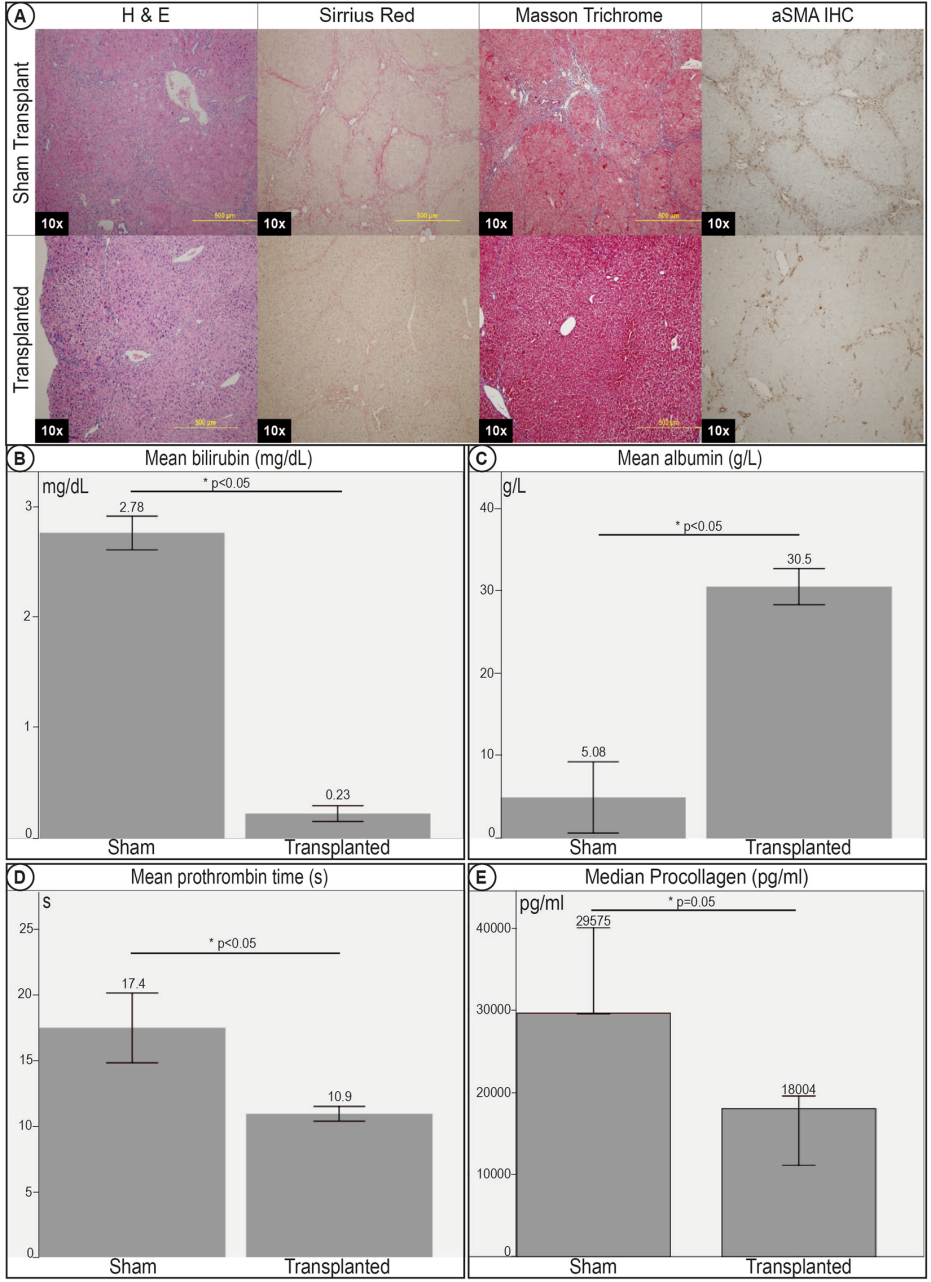
Improvement in histology and clinical correlates of liver cirrhosis in transplanted mice 2 months post –transplant. (**A**) Histology sections of sham transplant mice livers and HFH transplanted mice livers. Histology stains with H&E, Sirrius Red and Masson Trichrome demonstrated improvement of histological markers of cirrhosis, with less bridging fibrosis, architectural distortion and nodule formation in mice transplanted with HFH. IHC for aSMA demonstrated evidence of reduced myofibroblast activation in transplanted livers. Transplanted mice demonstrated improvements in clinical correlates of cirrhosis. There was a significant improvement in (**B**) hyperbiliruminemia, (**C**) hypoalbuminemia, and (**D**) coagulopathy. (**E**) Elisa for serum pro-collagen was significantly lower after transplant, demonstrating improvement in serum markers of fibrosis.

SR, MT, and H&E staining (Fig. 7A) showed consistently significant improvement with lower fibrosis and incomplete nodule formation as compared to mice not transplanted with HFH.

Quantitative image morphometry for areas occupied by SR staining and aSMA IHC further confirmed the improvement in cirrhosis in transplanted mice compared to sham transplant mice. ELISA for serum pro-collagen was also consistent (Fig. 7E), showing lower serum pro-collagen in transplanted mice compared to non-transplanted sham transplant mice.

### HFH transplantation demonstrated improvement of clinical correlates of liver cirrhosis

Significant improvement was seen in clinical correlates of cirrhosis in the mice post transplantation.

The hypoalbuminemia, coagulopathy, and hyperbilirubinemia were significantly better than mice who received sham transplants. The mean albumin level in transplanted mice was 30.5 g/L (28.2–33.5), while the mean albumin in sham transplant mice was 5.08 g/L (2.76–13.4), p < 0.05. The mean bilirubin was 0.23 mg/dL (0.15–0.31) in transplanted mice as compared to 2.78 mg/dL (2.60–2.96) in sham transplant mice, p < 0.05. The mean PT in transplanted mice was 10.9 s (10.1–11.5) as compared to 17.4 s (13.8–21.5) in sham transplant mice, p < 0.05. Of note, these levels seen in the transplanted mice were within normal ranges and were statistically insignificant when compared to similar age mice not given TA. (Fig. 7B–D)

There was no difference in the spleen-body weight ratio between transplanted and sham transplant mice. However, none of the transplanted mice showed detectable ascites or tumour formation

### Safety profile

We followed the transplanted mice for 6 months post-transplant (10 months administration of oral TA), and found no evidence to suggest malignant transformation. There were no nodules to suggest dysplasia or the development of hepatocellular carcinoma.

## Discussion

In this study, we report a murine model of liver cirrhosis that replicates the full clinical spectrum of cirrhosis, with structural changes of fibrosis and nodule formation, functional deficits as well as clinical correlates of portal hypertension, decompensation and oncogenesis. Cellular transplantation of human fetal progenitor cells successfully engrafts the cirrhotic liver, with reversal of fibrosis, improvement in liver function and resolution of decompensation. The immune-permissive model makes this a valuable tool for testing efficacy of human progenitor cells in treating end stage liver disease.

Stem/progenitor cell transplantation remains an extremely promising treatment modality for patients with end stage liver disease. Advances in the source options of candidate progenitor cells include those derived from induced pluripotent stem cells (iPSCs), embryonic stem cells, induced hepatocytes from fibroblasts and de novo progenitor cells from adult liver. While current efforts have focused on increasing repopulation, many of these high repopulation models are not clinically relevant as they do not replicate the spectrum of decompensated liver disease and do not demonstrate reversal.

Thioacetamide (TA) has been used for a long time to induce liver injury in rat and more recently mice^21^. This hepatotoxicity is achieved via conversion to toxic metabolites especially thioacetamide sulphoxide, which causes direct hepatotoxicity with centrilobular necrosis. This has been shown to progress to micronodular, and then macronodular cirrhosis^22^. It has also been shown to produce a stable liver fibrosis, which is similar in clinical progression to fibrosis in humans^15^. Youchev *et.al*. used a TA rat model and showed that they were able to achieve massive repopulation with allogenic transplantation of rat mature hepatocytes or stem/progenitor cells with improvement in fibrosis and decrease in transcript levels of fibrotic genes^23^. Our model goes one step further in demonstrating the efficacy of human progenitor cells in reversing complications of decompensated liver disease in an immune-permissive mouse. In our previous work, we have demonstrated that TA induces cirrhosis in animals, which can be used for cell testing^24^. In this paper, we further demonstrate that this protocol can further induce full clinical correlates of decompensated liver cirrhosis, which can be improved with cellular transplantation. This further highlights the value of this model to reflect the clinical needs. To our knowledge, no such mice model has hitherto, been reported.

To elicit clinical decompensation in our model, we administered TA to NSG mice and followed up the mice way beyond the typically reported 2–3 months. Indeed, the animals demonstrated the progressive development of fibrosis to nodular cirrhosis by 6 months of oral TA administration. Complications of cirrhosis ensued after 6 months, namely portal hypertension, ascites, hypoalbuminemia, coagulopathy, jaundice, encephalopathy, and oncogenesis. This replicates the full spectrum of decompensated liver disease in the human patient. We used NOD *scid gamma* mice that were T-cell, B-cell, NK-cell and complement deficient, and had defective macrophages and dendritic cells^25,26^. Several lines of work have demonstrated the critical role of NK cells in abrogating liver fibrosis^27^ and we postulated that the absence of NK cells may have accelerated the progression of fibrosis in this model. To confirm this, we repeated the experiments in C57BL/6 animals and while fibrosis was evident at similar time points, the degree and speed of cirrhosis development were indeed lower in the C57BL/6 mice compared to the NSG mice, although the indices were not numerically significant given the small numbers of animals.

HFH were chosen as they are the most physiological liver progenitor cells in the human, and would be ideal to test the model to see if it could be used to investigate cellular therapy. Cellular transplantation with the HFH cells not only showed improvement of liver fibrosis, but showed reversal in the clinical correlates of cirrhosis, providing principle of proof of efficacy in using such an approach to treat patients with end stage liver disease. This small rodent model will allow testing of efficacy and safety of other candidate progenitor cells as well as a large array of anti-fibrotic drugs, potentially accelerating drug development in preclinical studies. It will also be invaluable in allowing *in vivo* interrogation of the mechanism for fibrosis abrogation. In our model, we have tracked only the engraftment of hepatocytes. We clearly show the discordance between degree of engraftment of parenchymal cells, reversal of fibrosis and improvement in clinical outcomes. Presumably, the liver function may improve from contributions from the paracrine effect of non-parenchymal fractions, either by direct engraftment to normalise the microenvironment, or by indirect stimulation of regeneration.

In summary, we have demonstrated an immune-permissive murine model of liver cirrhosis that recapitulates the clinical manifestation of liver cirrhosis in humans. We believe this will be a valuable bridge that will accelerate the translational development of stem cells or anti-fibrotic therapy to impact patients with end stage liver disease.

## Data Availability

The datasets generated during and/or analysed during the current study are available from the corresponding author on reasonable request.
